# Flourishing in head and neck cancer survivors

**DOI:** 10.1002/cam4.4636

**Published:** 2022-03-11

**Authors:** Alexandria Harris, Jinhong Li, Karley Atchison, Christine Harrison, Daniel Hall, Tyler VanderWeele, Jonas T. Johnson, Marci L. Nilsen

**Affiliations:** ^1^ School of Medicine University of Pittsburgh Pittsburgh Pennsylvania USA; ^2^ Department of Otolaryngology University of Pittsburgh School of Medicine Pittsburgh Pennsylvania USA; ^3^ Department of Biostatistics, School of Public Health University of Pittsburgh Pittsburgh Pennsylvania USA; ^4^ Department of Surgery University of Pittsburgh Pittsburgh Pennsylvania USA; ^5^ Center for Health Equity Research and Promotion Veterans Affairs Pittsburgh Healthcare System Pittsburgh Pennsylvania USA; ^6^ Geriatric Research Educational and Clinical Center Veterans Affairs Pittsburgh Healthcare System Pittsburgh Pennsylvania USA; ^7^ Wolff Center UPMC Pittsburgh Pennsylvania USA; ^8^ Department of Epidemiology Harvard T. H. Chan School of Public Health Boston Massachusetts USA; ^9^ Human Flourishing Program, Institute for Quantitative Social Science Harvard University Cambridge Massachusetts USA; ^10^ Department of Acute and Tertiary Care, School of Nursing University of Pittsburgh Pittsburgh Pennsylvania USA

## Abstract

**Background:**

There is a growing cohort of head and neck cancer (HNC) patients affected by late‐ and long‐term posttreatment side effects. Our study evaluates the relationship between the demographics, clinical characteristics, and posttreatment symptom burden with the subjective sense of flourishing among HNC survivors.

**Methods:**

A cross‐sectional, single‐center study of adult survivors of squamous cell cancer of the oral cavity, oropharynx, and larynx/hypopharynx who completed the Secure Flourishing Index (SFI) and patient‐reported outcomes related to depression, anxiety, swallowing dysfunction, neck disability, and insomnia between November 2020 and April 2021.

**Results:**

A total of 100, predominantly male (86%), survivors with an average age of 63.0 ± 9.6 were included in the study. Univariable analysis showed a significant association between higher flourishing scores and advanced age (95% CI: [0.011, 0.84], *p* = 0.0441), normal diet (95% CI: [5.79, 31.18], *p* = 0.0149), employment (95% CI: [1.24, 17.20], *p* = 0.0239), higher income (95% CI: [7.30, 27.72], *p* = 0.0248), and decreased reported difficulty paying for needs (95% CI: [−33.46, −18.88], *p* < 0.001). Flourishing was inversely associated with higher symptoms of depression (95% CI: [−2.23, −1.15], *p* < 0.001), anxiety (95% CI: [−1.92,‐0.86], *p* < 0.001), swallowing dysfunction (95% CI: [−0.77, −0.26], *p* < 0.001), neck disability (95% CI: [−1.05, −0.35], *p* < 0.001), and insomnia (95% CI: [−1.12, −0.22], *p* = 0.004) in the multivariable analysis.

**Conclusions:**

Common late‐ and long‐term side effects of HNC treatment and financial hardship are associated with lower levels of flourishing or a more negative perception of life after treatment. Results highlight the importance of symptom burden for survivors' overall evaluation of their quality of life.

## Graphical abstract

In this prospective, cross‐sectional study of 100 cancer survivors, symptoms of depression, anxiety, swallowing dysfunction, neck disability, insomnia along with perceived difficulty paying for needs was found to have a significant impact on human flourishing or the survivor’s perception of well‐being. In head and neck cancer survivors, human flourishing is associated with a higher symptom burden and financial toxicity.

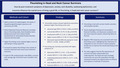

## 1 INTRODUCTION

Head and neck cancer (HNC) survivorship has become a critical issue within head and neck oncology. Increasingly, intensified treatment regimens and a younger patient demographic have contributed to a large survivorship population with long posttreatment courses.^1^, ^2^, ^3^, ^4^, ^5^ With these improvements in survival and increased treatment intensity, patients experience late‐ and long‐term side effects of their disease and treatment.^6^, ^7^, ^8^, ^9^, ^10^, ^11^ The diagnosis of cancer itself has been associated with adverse effects such as posttraumatic stress symptoms, complicated grief, depression, and anxiety.^12^, ^13^, ^14^, ^15^ These patients then face the sequela of treatment, including mucositis, infections, nausea, hair loss, fatigue, and weight loss.^16^, ^17^ After treatment, survivors face complicated posttreatment courses including long‐standing treatment‐related toxicities such as fibrosis, neck disability, swallowing dysfunction, dental decay, and change in physical appearance.^18^ This complicated course affects the well‐being and quality of life of patients.

Survivors manage complex, progressive side effects of treatment throughout their lives. For many patients, side effects of treatment contribute to the inability to work, drive, eat with their family, or communicate with friends, impacting their physical, mental, and social health as well.^19^, ^20^ Studies have shown relationships between outcomes, such as swallowing dysfunction and neck disability, the prevalence of pain, symptom burden, anxiety, depression, and the burden of treatment, though how these symptoms affect the general satisfaction of survivors is poorly understood.^16^, ^21^, ^22^, ^23^

Flourishing is a sense of well‐being defined as living in a “state in which all aspects of a person's life are good.”^24^ The recently validated Flourishing Index (FI) assesses 5 central domains of a good life: (1) happiness and life satisfaction, (2) meaning and purpose, (3) character and virtue, (4) close social relationships, and (5) mental and physical health. The Secure Flourishing Index (SFI) adds a sixth domain related to financial and material stability.^24^ By measuring more than mere mental and physical health, the assessment of flourishing can quantify unrecognized adverse impacts of healthcare treatments while simultaneously elucidating how aspects of patients’ lives may continue to flourish even as physical health declines.^25^ As such the assessment of flourishing is ideally suited for HNC survivorship initiatives that appropriately focus on treatment‐related toxicities and their profound impact on quality of life. Although recent studies have quantified important clinical outcomes and symptoms following HNC treatment,^16^, ^26^, ^27^ they fail to capture how those symptoms impact other domains of flourishing beyond mere mental and physical health. This study fills this gap by assessing flourishing in a cohort of HNC survivors, quantifying the extent to which individuals live within an “optimal range of functioning” with the “promotion of goodness, growth, and resilience.” We examine the relationship between flourishing, demographics, clinical characteristics, and posttreatment symptoms to better understand how HNC treatment affects survivors’ lives.

## 2 METHODS

We conducted a cross‐sectional survey of 100 HNC survivors who completed the SFI and patient‐reported outcome (PROs) questionnaires related to neck disability, anxiety, depression, insomnia, and swallowing dysfunction prior to evaluation in the multidisciplinary UPMC Head and Neck Cancer survivorship clinic between November 2020 and April 2021. All adults (≥18 years) who completed treatment for squamous cell carcinoma of the oral cavity, oropharynx, and larynx/hypopharynx and who had completed treatment at the time of data collection were eligible. Exclusion criteria included a history of recurrence, secondary primary carcinomas, or distant metastasis. University of Pittsburgh Institutional Review Board approved the study: STUDY20070027.

### 2.1 Demographics and clinical characteristics

Demographics were collected from the survivors at the time of the survey with clinical characteristics abstracted from the medical record. Variables obtained included: age, self‐reported sex, and race (White, Other [e.g., African American/Asian]), living with or without a partner, tumor site, American Joint Committee on Cancer (7th and 8th Edition) staging (i.e., Tis‐II and III/IV), treatment modality (i.e., surgery alone, nonsurgical, and surgery plus adjuvant), and time since treatment completion. Functional Oral Intake Scale (FOIS) data were collected and categorized into three groups: tube feed‐dependent (FOIS score 1–3), oral intake limitations (FOIS score 4–6), and normal diet (FOIS score 7).^28^

### 2.2 Flourishing index

Flourishing was assessed using the SFI, a 12‐question measure divided into six 2‐question domains: happiness and life satisfaction (domain 1), mental and physical health (domain 2), meaning and purpose (domain 3), character and virtue (domain 4), close social relationships (domain 5), and financial and material stability (domain 6).^29^, ^30^ Analysis was performed using responses from the SFI and the FI, which excludes domain 6 on financial stability. Each domain is scored from 1 to 10, with the total scores averaged by the number of items. The scores on the FI and SFI range from 0 to 100 and 0 to 120, respectively, with higher scores indicating a life in which all aspects are considered good. The SFI and FI have been used to measure flourishing in other settings with high validity and reliability (FI *α* = 0.89, SFI *α* = 0.86).^29^

### 2.3 Patient Health Questionaire‐8

The Patient Health Questionnaire eight‐item depression scale (PHQ8) is a valid and reliable index used to assess the prevalence and severity of depression in a population.^31^, ^32^ The PHQ8 consists of questions on eight of the nine criteria for DSM‐V diagnosis of depressive disorders with questions on self‐harm or suicidal ideation omitted due to inability to provide adequate intervention at the time of the survey, patients report a positive response less commonly with scoring thresholds remaining similar with and without this question.^33^, ^34^ The PHQ8 scoring range is 0–24 and the minimally clinically important difference (MCID) is 3 points.^35^ For this study, scores were evaluated continuously, with higher scores indicating more severe depressive symptoms.

### 2.4 Generalized Anxiety Disorder‐7

The Generalized Anxiety Disorder seven‐item anxiety scale (GAD7) is related to DSM‐V generalized anxiety criteria. The questionnaire is valid with strong test–retest reliability with sensitivity to treatment response.^36^, ^37^ The total score ranges from 0 to 21 with an MCID of 4 points and higher scores indicating more severe anxiety symptoms.^38^ Scores were then evaluated continuously with flourishing to examine the relationship.

### 2.5 Neck Disability Index

The Neck Disability Index is a 10‐question measure of disability resulting from neck pain, with higher scores indicating more severe disability.^39^ Questions evaluate the impact pain has on functional activities such as personal care, sleep, and movement. Each item is scored from 0 to 5, increasing with severity, with scale scores ranging from 0 to 50. MCID is reported in prior studies as approximately 10 points.^40^, ^41^, ^42^, ^43^ The NDI has been used to measure neck dysfunction reliably and consistently in HNC patients.^39^, ^44^, ^45^, ^46^, ^47^, ^48^ In our study, NDI was used as a continuous scale to evaluate the relationship with flourishing, with higher scores indicating increasingly severe neck impairment.

### 2.6 Eating Assessment Tool

The Eating Assessment Tool (EAT)‐10 questionnaire, a 10‐question symptom‐specific measure of symptoms of dysphasia, was used to assess swallowing dysfunction. The EAT‐10 questionnaire has shown reliability and internal consistency in HNC patients.^49^, ^50^ Total scores range from 0 to 40, with a score of 3 or more indicating swallowing dysfunction. Scores greater than 15 have been demonstrated to have good specificity (70.6%) in predicting aspiration.^51^ Since most HNC patients experience some level of swallowing dysfunction, scores were analyzed on a continuous scale with higher values indicating more severe dysfunction.^50^

### 2.7 Insomnia severity index

Insomnia was measured using the insomnia severity index (ISI), a seven‐item questionnaire with questions on sleep quality, maintenance, and interference with daily functioning. It is a reliable and valid questionnaire with questions regarding the past 2 weeks.^52^, ^53^ Total score ranges from 0 to 28, and measurements used as a continuous scale with higher scores indicating more clinically severe insomnia.

### 2.8 Statistical analysis

All statistical analysis was performed using RStudio (1.1.456; RStudio, Inc) and SAS v9.4 (SAS Institute). We calculated frequency (percentage) for categorical variables and mean ± standard deviation (SD) for continuous variables for the descriptive analysis. Univariable linear regression was performed to analyze the association between flourishing scores, subdomains, and independent variables, including age, time since treatment, sex, race, marital status, FOIS, American Joint Committee on Cancer (AJCC) stage (i.e., early [I/II] or advanced [III/IV]), tumor site (oral cavity, oropharynx, and larynx/hypopharynx), HPV, education, occupation, income level, difficulty to pay, and scores of common quality‐of‐life indices including PHQ8, GAD7, EAT10, NDI, and ISI. Considering both statistical and clinical significance, the multivariable linear regression models with the SFI included individual PRO (PHQ8, GAD7, EAT10, NDI, and ISI), age, time since treatment, sex, race, AJCC staging, cancer site, education, difficulty paying for needs. FOIS was excluded from the analysis of depression, swallowing dysfunction, and neck disability due to high collinearity with the PRO. FOIS was included in the analysis of anxiety and insomnia. Variables with a *p* value of less than 0.05 were considered significant.

## 3 RESULTS

One hundred, predominantly White (*n* = 88, 88%), male (*n* = 86, 86%) survivors with an average age of 63.0 ± 9.6 years qualified for the study, completed the SFI, and were included in the final analysis. Demographics and clinical characteristics are summarized in Table 1. Oropharyngeal cancers were the most common site (*n* = 54, 54%) followed by oral cavity (*n* = 26, 26%) and larynx/hypopharynx (*n* = 20, 20%). Of the 54 survivors with oropharyngeal cancer, 51 (94.4%) were HPV associated. The majority of patients were treated for advanced disease (*n* = 66, 66%) compared with early stage (*n* = 32, 32%) with two survivors having an unknown stage at diagnosis. Treatment consisted of surgical intervention alone (*n* = 6, 6%), surgery plus adjuvant chemo‐ and/or radiotherapy (*n* = 57, 57%), and nonsurgical intervention (*n* = 37, 37%) and average time since treatment was 42.6 ± 70.4 months. Most survivors had an FOIS score between 4 and 6 (*n* = 61, 61%), indicating a modified, nontube‐dependent diet followed by a nonmodified diet (*n* = 20, 20%) and tube‐dependent nutrition (*n* = 18, 18%).

**TABLE 1:** Demographic and clinical characteristics

	Mean ± SD	*n* (%)
Age (years)	63.0 ± 9.6	
Time Since Treatment (months)^a^	42.6 ± 70.4	
Living with partner		
No		30 (30)
Yes		70 (70)
Sex		
Male		86 (86)
Female		14 (14)
Race		
White		88 (88)
Other		12 (12)
Functional Oral Intake Scale (FOIS)		
Tube Feed‐Dependent (FOIS 1–3)		18 (18)
Oral Intake Limitations (FOIS 4–6)		61 (62)
Normal Diet		20 (20)
Site		
Oral Cavity		26 (26)
Oropharynx		54 (54)
Larynx/Hypopharynx		20 (20)
Stage		
Early		32 (33)
Advanced		66 (67)
Treatment		
Surgery Alone		6 (6)
Surgery + Adjuvant		57 (57)
Nonsurgical		37 (37)
HPV		
Positive		51 (53)
Negative		5 (5)
N/A		40 (42)
Education		
Some high school, diploma, or GED		34 (34)
Some College, Associates, Bachelors		49 (49)
Graduate		16 (16)
Occupation		
Not Working		48 (48)
Working		51 (51)
Income		
0–20 k		28 (28)
20–99 k		42 (42)
100 k+		29 (29)
Difficulty paying for needs		
Not difficult at all		70 (71)
Somewhat or extremely difficult		29 (29)

^a^ Median is 12.5 months.

The SFI results were evaluated by domains and included both the 10‐domain FI, with scores ranging from 27 to 100 and a mean of 79.3 ± 16.6, and 12‐domain SFI with scores ranging from 29 to 120 and a mean of 95.6 ± 20.3. The relationships between flourishing scores, subdomain scores, and demographic and clinical characteristics are summarized in Table 2. Univariable analysis showed higher flourishing scores were associated with advanced age (0.43, 95% CI: [0.011, 0.84], *p* = 0.0441), normal diet (18.48, 95% CI: [5.79, 31.18], *p* = 0.0149), employment (9.22, 95% CI: [1.24, 17.20], *p* = 0.0239), higher income (17.51, 95% CI: [7.30, 27.72], 0.0248), and decreased reported difficulty paying for needs such as food, income, housing, and healthcare (26.17, 95% CI: [−33.46, −18.88], *p* <0.001). The FI was used to examine the relationship between financial toxicity and flourishing. Individuals who were not working due to unemployment, disability, or retirement had an average FI score of 75.8 ± 17.1, significantly lower than those currently working (82.7 ± 15.8, *p* = 0.0395). Similarly, flourishing scores increased (*p* = 0.0248) as annual income increased from $0 to $20,000 (72.5 ± 20.2), $21,000 to $99,000 (81.9 ± 15.1), and those making over $100,000 (82.8 ± 11.7). The survivors who reported some or high levels of difficulty paying for basic needs reported lower flourishing scores(65.4 ± 19.2) than those who reported having no difficulty (85.1 ± 11.4, *p* < 0.001), an effect present across all subdomains as shown in Table 3.

**TABLE 2:** Univariable analysis of demographic and clinical characteristics with overall FI and SFI

	Flourishing Index (5 Domains)			Secure Flourishing Index (6 Domains)		
	Mean ± SD	Coefficient (95% CI)	*p* value^a^	Mean ± SD	Coefficient (95% CI)	*p* value^a^
Age (years)		0.34 (−0.0019, 0.68)	0.0513		0.43 (0.011, 0.84)	0.0441
Time Since Treatment (months)		0.041 (−0.0060, 0.087)	0.0868		0.050 (−0.0073, 0.11)	0.0868
Living with partner						
No	78.1 ± 17.1	Base	0.638	92.9 ± 21.3	Base	0.402
Yes	79.8 ± 16.5	1.72 (−5.52, 8.95)		96.7 ± 20.0	3.74 (−5.08, 12.55)	
Sex						
Male	79.1 ± 16.7	Base	0.767	95.4 ± 20.2	Base	0.863
Female	80.5 ± 17.0	1.43 (−8.13, 10.99)		96.4 ± 21.8	1.02 (10.66, 12.71)	
Race						
White	79.1 ± 16.7	Base	0.26	95.4 ± 20.2	Base	0.171
Other	74.2 ± 23.0	−5.80 (−15.95, 4.35)		88.0 ± 30.0	−8.58 (−20.94, 3.78)	
Functional Oral Intake Scale (FOIS)						
Tube Feed‐Dependent	72.7 ± 17.8	Base	0.0188	87.2 ± 21.5	Base	0.0149
(FOIS 1–3)						
Oral Intake Limitations (FOIS 4–6)	78.5 ± 16.5	5.48 (−3.12, 14.09)		94.7 ± 20.2	7.02 (−3.46, 17.50)	
Normal Diet	87.8 ± 12.8	14.69 (4.27, 25.12)		106.2 ± 15.3	18.48 (5.79, 31.18)	
Site						
Oral Cavity	76.5 ± 19.2	Base	0.608	91.8 ± 23.1	Base	0.518
Oropharynx	80.4 ± 15.0	3.85 (−4.07, 11.78)		97.3 ± 18.1	5.51 (−4.16, 15.18)	
Larynx/Hypopharynx	81.3 ± 18.2	3.45 (−6.43, 13.33)		98.2 ± 22.4	4.03 (−8.01, 16.08)	
Stage						
Early	82.1 ± 18.3	Base	0.215	99.5 ± 21.8	Base	0.155
Advanced	77.6 ± 15.9	−4.49 (−11.63, 2.66)		93.2 ± 19.6	−6.27 (−14.97, 2.42)	
Treatment						
Surgery Alone	78.5 ± 20.7	Base	0.835	94.7 ± 25.6	Base	0.808
Surgery + Adjuvant	78.5 ± 16.2	0.0088 (−14.29, 14.30)		94.5 ± 20.2	−0.14 (−17.60, 17.32)	
Nonsurgical	80.6 ± 17.1	2.07 (−12.59, 16.73)		97.3 ± 20.1	2.60 (−15.30, 20.51)	
HPV						
Positive	79.1 ± 16.6	Base	0.481	95.8 ± 19.6	Base	0.373
Negative	87.6 ± 7.1	8.52 (−7.06, 24.10)		106.6 ± 8.4	10.82 (−8.159, 29.79)	
N/A	77.3 ± 17.8	−1.10 (−8.03, 5.82)		92.4 ± 22.4	−2.55 (−10.98, 5.89)	
Education						
Some high school, diploma, or GED	78.7 ± 19.2	Base	0.321	94.2 ± 24.2	Base	0.512
Some College, Associates, Bachelors	81.4 ± 15.6	−4.36 (−4.70, 10.08)		97.9 ± 18.9	3.62 (−5.46, 12.70)	
Graduate degree	74.4 ± 14.0	−4.36 (−14.40, 5.68)		91.6 ± 15.8	−2.61 (12.70, 9.72)	
Occupation						
Not Working	75.8 ± 17.1	Base	0.0395	90.9 ± 21.1	Base	0.0239
Working	82.7 ± 15.8	6.89 (0.34, 13.45)		100.1 ± 18.9	9.22 (1.24, 17.20)	
Income						
0–20 k	72.5 ± 20.2	Base	0.0248	85.1 ± 25.5	Base	0.001
20–99 k	81.9 ± 15.1	8.34 (0.48, 16.21)		99.2 ± 17.5	12.57 (3.17, 21.97)	
100 k+	82.8 ± 11.7	11.47 (2.93, 20.01)		101.6 ± 12.8	17.51 (7.30, 27.72)	
Difficulty paying for needs						
Not difficult at all	85.1 ± 11.4	Base	<0.001	103.3 ± 13.3	Base	<0.001
Somewhat or extremely difficult	65.4 ± 19.2	−19.68 (−25.88, −13.48)		77.1 ± 22.9	−26.17 (−33.46, −18.88)	

^a^ *p* value according to Linear Regression Model and Likelihood Ratio Test; significance level at *p* < 0.05.

**TABLE 3:** Demographic and clinical characteristics significant subdomain analysis

	Domain 1 (Happiness and Life Satisfaction)			Domain 2 (Mental and Physical Health)		
	Mean ± SD	Coefficient (95% CI)	*p* value^a^	Mean ± SD	Coefficient (95% CI)	*p* value^a^
Age (years)	0.05 (0.009, 0.10)	0.0177		0.0285 (−0.02, 0.08)	0.212	
Time Since Treatment (months)	0.015 (0.002, 0.013)	0.0126		0.007 (0, 0.01)	0.0219	
Functional Oral Intake Scale (FOIS)						
Tube Feed‐Dependent (FOIS 1–3)	6.6 ± 2.1	Base	0.0162	6.0 ± 2.5	Base	0.00116
Oral Intake Limitations (FOIS 4–6)	7.5 ± 2.1	0.88 (−0.23, 1.99)		7.0 ± 2.1	1.04 (−0.06, 2.14)	
Normal Diet	8.6 ± 2.0	1.96 (0.63, 3.31)		8.5 ± 1.5	2.48 (1.15, 3.82)	
Occupation						
Not Working	7.0 ± 2.3	Base	0.0100	6.6 ± 2.3	Base	0.0178
Working	8.1 ± 1.9	1.10 (0.27, 1.93)		7.6 ± 2.1	1.035 (0.19, 1.89)	
Income						
0–20 k	6.5 ± 2.6	Base	0.00545	6.0 ± 2.5	Base	0.00211
20–99 k	7.8 ± 1.9	1.28 (0.29, 2.28)		7.4 ± 2.0	1.45 (0.45, 2.46)	
100 k+	8.2 ± 1.7	1.69 (0.61, 2.77)		7.8 ± 1.9	1.84 (0.76, 2.94)	
Difficulty paying for needs						
Not difficult at all	8.3 ± 1.6	Base	<0.001	7.9 ± 1.6	Base	<0.001
Somewhat or extremely difficult	5.8 ± 2.3	−2.54 (−3.34, −1.75)		5.2 ± 2.3	−2.68 (−3.48, −1.89)	

	Domain 3 (Meaning and Purpose)			Domain 4 (Character and Virtue)		
	Mean ± SD	Coefficient (95% CI)	*p* value^a^	Mean ± SD	Coefficient (95% CI)	*p* value^a^
Age (years)	0.0395 (0, 0.08)	0.0419		0.011 (−0.02, 0.04)	0.527	
Time Since Treatment (months)	0.00315 (0, 0.01)	0.242		0.0007 (0, 0.01)	0.761	
Functional Oral Intake Scale (FOIS)						
Tube Feed Dependent (FOIS 1–3)	7.8 ± 2.2	Base	0.0959	7.8 ± 2.1	Base	0.070
Oral Intake Limitations (FOIS 4–6)	8.2 ± 1.9	0.445 (−0.54, 1.43)		8.4 ± 1.7	0.54 (−0.31, 1.4)	
Normal Diet	9.0 ± 1.5	1.25 (0.06, 2.44)		9.0 ± 0.9	1.20 (0.16, 2.23)	
Occupation						
Not Working	7.9 ± 2.1	Base	0.0303	8.3 ± 1.8	Base	0.450
Working	8.7 ± 1.6	0.82 (0.08, 1.55)		8.5 ± 1.6	0.25 (−0.41, 0.91)	
Income						
0–20 k	7.6 ± 2.6	Base	0.0464	8.3 ± 2.1	Base	0.480
20–99 k	8.5 ± 1.6	0.86 (0.03, 1.75)		8.3 ± 1.5	0.03 (−0.75, 0.83)	
100 k+	8.7 ± 1.4	1.16 (0.19, 2.12)		8.7 ± 1.4	0.46 (−0.41, 1.32)	
Difficulty paying for needs						
Not difficult at all	8.9 ± 1.2	Base	<0.001	8.7 ± 1.3	Base	0.00324
Somewhat or extremely difficult	6.8 ± 2.5	−2.12 (−2.83, −1.41)		7.7 ± 2.2	−1.05 (−1.74, −0.36)	

	Domain 5 (Close Social Relationships)			Domain 6 (Financial and Material Stability)		
	Mean ± SD	Coefficient (95% CI)	*p* value^a^	Mean ± SD	Coefficient (95% CI)	*p* value^a^
Age (years)		0.0375 (0, 0.08)	0.0603		0.044 (−0.01, 0.1)	0.209
Time Since Treatment (months)		0.00185 (0, 0.01)	0.497		0.0045 (0, 0.01)	0.0921
Functional Oral Intake Scale (FOIS)						
Tube Feed Dependent (FOIS 1–3)	8.4 ± 2.1	Base	0.433	7.3 ± 2.8	Base	0.0542
Oral Intake Limitations (FOIS 4–6)	8.3 ± 1.9	−0.17 (−1.19, 0.85)		8.1 ± 2.6	0.77 (−0.54, 2.08)	
Normal Diet	8.9 ± 1.8	0.46 (−0.77, 1.69)		9.2 ± 1.5	1.89 (0.31, 3.48)	
Occupation						
Not Working	8.3 ± 2.0	Base	0.521	7.5 ± 2.8	Base	0.0201
Working	8.5 ± 1.9	0.25 (−0.52, 1.02)		8.7 ± 2.1	1.16 (0.19, 2.15)	
Income						
0–20 k	8.0 ± 2.5	Base	0.409	6.4 ± 3.2	Base	<0.001
20–99 k	8.6 ± 1.6	0.56 (−0.38, 1.49)		8.5 ± 2.1	2.12 (1.03, 3.20)	
100 k+	8.6 ± 1.8	0.58 (−0.43, 1.6)		9.4 ± 1.0	3.02 (1.85, 4.20)	
Difficulty paying for needs						
Not difficult at all	8.8 ± 1.5	Base	<0.001	9.1 ± 1.6	Base	<0.001
Somewhat or extremely difficult	7.4 ± 2.5	−1.45 (−2.25, −0.66)		5.9 ± 2.9	−3.24 (−4.14, −2.35)	

^a^ *p* value according to Linear Regression Model and Likelihood Ratio Test; significance level at *p* < 0.05.

The reported PRO scores (PHQ8, GAD7, EAT10, NDI, and ISI) were compared with the flourishing scores reported in Table 4. With each PRO, higher scores indicate increasingly severe symptoms. Decreases in the flourishing score were associated with a higher symptom burden of depression, anxiety, swallowing dysfunction, neck disability, and insomnia (*p* < 0.001). Depression, anxiety, and swallowing dysfunction showed decreased flourishing across all six subdomains, whereas neck disability and insomnia showed significant decreases across all subdomains except domain 4 (character and virtue).

**TABLE 4:** Univariable analysis of patient‐reported symptoms with flourishing and subdomain scores

	Mean ± SD	Flourishing Index (5 Domains)		Secure Flourishing Index (6 Domains)	
		Coefficient (95% CI)	*p* value^a^	Coefficient (95% CI)	*p* value^a^
Depression (PHQ8)	7.4 ± 6.0	−2.13 (−2.59, −1.66)	<0.001	−2.64 (−3.20, −2.08)	<0.001
Anxiety (GAD7)	4.8 ± 6.2	−1.76 (−2.25, −1.27)	<0.001	−2.22 (−2.81, −1.63)	<0.001
Swallowing (EAT10)	15.4 ± 11.8	−0.61 (−0.86, −0.36)	<0.001	−0.76 (−1.07, −0.45)	<0.001
Neck Disability (NDI)	10.2 ± 9.3	−0.94 (−1.25, −0.63)	<0.001	−1.20 (−1.57, −0.83)	<0.001
Insomnia (ISI)	7.8 ± 7.1	−1.08 (−1.50, −0.66)	<0.001	−1.35 (−1.86, −0.84)	<0.001

	Domain 1 (Happiness and Life Satisfaction)		Domain 2 (Mental and Physical Health)		Domain 3 (Meaning and Purpose)	
	Coefficient (95% CI)	*p* value^a^	Coefficient (95% CI)	P value^a^	Coefficient (95% CI)	*p* value^a^
Depression (PHQ8)	−0.28 (−0.34, −0.23)	<0.001	−0.31 (−0.37, −0.26)	<0.001	−0.20 (−0.26, −0.14)	<0.001
Anxiety (GAD7)	−0.24 (−0.31, −0.19)	<0.001	−0.26 (−0.32, −0.21)	<0.001	−0.17 (−0.23, −0.11)	<0.001
Swallowing (EAT10)	−0.085 (−0.12, −0.05)	<0.001	−0.11 (−0.14, −0.07)	<0.001	−0.050 (−0.08, −0.02)	0.00135
Neck Disability (NDI)	−0.14 (−0.17, −0.1)	<0.001	−0.15 (−0.19, −0.11)	<0.001	−0.080 (−0.12, −0.04)	<0.001
Insomnia (ISI)	−0.16 (−0.21, −0.11)	<0.001	−0.16 (−0.22, −0.12)	<0.001	−0.085 (−0.14, −0.03)	<0.001

	Domain 4 (Character and Virtue)		Domain 5 (Close Social Relationships)		Domain 6 (Financial and Material Stability)	
	Coefficient (95% CI)	*p* value^a^	Coefficient (95% CI)	*p* value^a^	Coefficient (95% CI)	*p* value^a^
Depression (PHQ8)	−0.090 (−0.15, −0.03)	0.00372	−0.19 (−0.25, −0.12)	<0.001	−0.26 (−0.34, −0.18)	<0.001
Anxiety (GAD7)	−0.065 (−0.12, −0.01)	0.0325	−0.15 (−0.21, −0.08)	<0.001	−0.23 (−0.31, −0.15)	<0.001
Swallowing (EAT10)	−0.033 (−0.06, −0.01)	0.0177	−0.035 (−0.07, 0.00)	0.0266	−0.075 (−0.12, −0.04)	<0.001
Neck Disability (NDI)	−0.033 (−0.07, 0.00)	0.0692	−0.075 (−0.12, −0.04)	<0.001	−0.13 (−0.18, −0.08)	<0.001
Insomnia (ISI)	−0.036 (−0.08, 0.01)	0.124	−0.10 (−0.15, −0.05)	<0.001	−0.14 (−0.21, −0.07)	<0.001

^a^ *p* value according to Linear Regression Model and Likelihood Ratio Test; significance level at *p* <0.05.

Multivariable linear regression analysis was performed for all PROs, which found persistence of univariable effect sizes. Regression demonstrated an inverse relationship between all measured PROs and secure flourishing scores. For each increase in PHQ8 score, flourishing was decreased by 1.69 points (95% CI: [−2.23, −1.15], *p* < 0.001), a clinically significant increase in depression score resulted in a decrease of flourishing by 5.07 points. GAD7 scores showed a decrease in the flourishing of 1.39 points (95% CI: [−1.92,‐0.86], *p* < 0.001). Each EAT10 point increase was associated with a 0.52 drop in the flourishing score (95% CI: [−0.77, −0.26], *p* < 0.001). NDI increase was associated with a 0.70‐point reduction in the flourishing score (95% CI: [−1.05, −0.35], *p* = 0.001). Insomnia was associated with a 0.67‐point decline in the flourishing score (95% CI: [−1.12, −0.22], *p* = 0.004). Regression also demonstrated a decrease in the flourishing scores by 14–21 points when survivors reported difficulty paying for needs across all models (*p* < 0.001). Final regression models are summarized in Table 5.

**TABLE 5:** Results of the multivariable linear regression between Each PRO and Secure Flourishing Index score. (A) Result of flourishing and depression multivariable analysis. (B) Result of flourishing and anxiety multivariable analysis. (C) Result of flourishing and swallowing dysfunction multivariable analysis. (D) Result of flourishing and neck disability multivariable analysis. (E) Result of flourishing and insomnia multivariable analysis

Variables	Coefficient (95% CI)	*p* value^a^
**A. Flourishing and Depression Multivariable Analysis**		
Intercept	91.24 (71.35, 111.13)	
Depression (PHQ‐8 Score)	−1.69 (−2.23, −1.15)	<0.001
Age	0.03 (−0.26, 0.31)	0.859
Time since treatment completion (months)	0 (−0.04, 0.04)	0.986
Sex		
Male	Base	0.090
Female	7.53 (−1.21, 16.26)	
Race		
White	Base	0.695
Other	0.99 (−7.49, 9.47)	
AJCC Stage		
Tis‐II^b^	Base	0.413
III/IV	−1.13 (−6.89, 4.63)	
Site		
Oral Cavity	Base	
Oropharynx	2.65 (−4.79, 10.1)	0.676
Larynx/Hypopharynx	4.11 (−4.31, 12.52)	0.703
Education		
Some high school, diploma, or GED	Base	
Some College, Associates, Bachelors	−2.78 (−8.67, 3.12)	0.538
Graduate Degree	−7.85 (−16.39, 0.7)	0.260
Difficulty Paying For Needs		
None	Base	<0.001
Some or extreme difficulty	−12.31 (−19.11, −5.51)	
**B. Flourishing and Anxiety Multivariable Analysis**		
Intercept	91.8 (68.21, 115.38)	
Anxiety (GAD7 Score)	−1.39 (−1.92, −0.86)	<0.001
Age	−0.05 (−0.36, 0.26)	0.770
Time since treatment completion (months)	0.01 (−0.03, 0.06)	0.586
Sex		
Male	Base	0.128
Female	6.98 (−2.06, 16.02)	
Race		
White	Base	0.596
Other	−2.28 (−10.81, 6.25)	
AJCC Stage		
Tis‐II^b^	Base	0.976
III/IV	−0.09 (−6.17, 5.98)	
Site		
Oral Cavity	Base	
Oropharynx	1.57 (−6.15, 9.3)	0.686
Larynx/Hypopharynx	2.25 (−6.6, 11.1)	0.614
Education		
Some high school, diploma, or GED	Base	
Some College, Associates, Bachelors	−4.21 (−10.48, 2.05)	0.184
Graduate Degree	−12.54 (−20.94, −4.14)	0.004
Difficulty Paying For Needs		
None	Base	<0.001
Some or extreme difficulty	−14.04 (−20.74, −7.35)	
Functional Oral Intake Scale (FOIS)		
Tube Feed Dependent (FOIS 1–3)	Base	
Oral Intake Limitations (FOIS 4–6)	2.74 (−4.57, 10.05)	0.458
Normal Diet (FOIS 7)	3.52 (−5.87, 12.92)	0.458
**C. Flourishing and Swallowing Multivariable Analysis**		
Intercept	96.99 (74.42, 119.56)	
Swallowing (Eat‐10 Score)	−0.52 (−0.77, −0.26)	<0.001
Age	−0.09 (−0.41, 0.23)	0.573
Time since treatment completion (months)	0.01 (−0.03, 0.06)	0.573
Sex		
Male	Base	0.038
Female	10.05 (0.59, 19.51)	
Race		
White	Base	0.159
Other	−6.28 (−15.07, 2.52)	
AJCC Stage		
Tis‐II^b^	Base	0.655
III/IV	−1.43 (−7.78, 4.92)	
Site		
Oral Cavity	Base	
Oropharynx	3.65 (−4.33, 11.62)	0.366
Larynx/Hypopharynx	1.68 (−7.51, 10.86)	0.718
Education		
Some high school, diploma, or GED	Base	
Some College, Associates, Bachelors	−1.48 (−7.92, 4.97)	0.650
Graduate Degree	−7.96 (−16.87, 0.95)	0.079
Difficulty Paying For Needs		
None	Base	<0.001
Some or extreme difficulty	−15.97 (−22.71, −9.23)	
**D. Flourishing and Neck Disability Multivariable Analysis**		
Intercept	89.96 (68.04, 111.88)	
Neck Disability (NDI Score)	−0.70 (−1.05, −0.35)	0.001
Age	−0.020 (−0.34, 0.29)	0.879
Time since treatment completion (months)	0.010 (−0.040, 0.050)	0.708
Sex		
Male	Base	0.031
Female	10.78 (1.00, 20.56)	
Race		
White	Base	0.428
Other	−3.52 (−12.3, 5.26)	
AJCC Stage		
Tis‐II^b^	Base	0.920
III/IV	−0.32 (−6.63, 5.99)	
Site		
Oral Cavity	Base	
Oropharynx	4.52 (−3.40, 12.45)	0.260
Larynx/Hypopharynx	5.61 (−3.57, 14.80)	0.228
Education		
Some high school, diploma, or GED	Base	
Some College, Associates, Bachelors	−2.21 (−8.70, 4.28)	0.501
Graduate Degree	−10.17 (−18.90, −1.44)	0.023
Difficulty Paying For Needs		
None	Base	<0.001
Some or extreme difficulty	−14.38 (−21.62, −7.15)	
**E. Flourishing and Insomnia Multivariable Analysis**		
Intercept	90.92 (64.37, 117.47)	
Insomnia (ISI Score)	−0.67 (−1.12, −0.22)	0.004
Age	−0.07 (−0.42, 0.28)	0.693
Time since treatment completion (months)	0.00 (−0.040, 0.050)	0.857
Sex		
Male	Base	0.090
Female	6.71 (−3.46, 16.88)	
Race		
White	Base	0.508
Other	−3.14 (−12.52, 6.25)	
AJCC Stage		
Tis‐II^b^	Base	0.513
III/IV	−0.20 (−6.90, 6.50)	
Site		
Oral Cavity	Base	
Oropharynx	2.45 (−6.09, 10.99)	0.569
Larynx/Hypopharynx	3.07 (−6.70, 12.84)	0.534
Education		
Some high school, diploma, or GED	Base	
Some College, Associates, Bachelors	−1.22 (−8.07, 5.62)	0.723
Graduate Degree	−9.21 (−18.67, 0.26)	0.056
Difficulty Paying For Needs		
None	Base	<0.001
Some or extreme difficulty	−16.17 (−23.51, −8.82)	
Functional Oral Intake Scale (FOIS)		
Tube Feed Dependent (FOIS 1–3)	Base	
Oral Intake Limitations (FOIS 4–6)	2.29 (−5.81, 10.39)	0.576
Normal Diet (FOIS 7)	0.00 (−6.31, 14.83)	0.425

*Note*: FOIS was removed from regression model in depression, swallowing, and neck disability due to high collinearity.

^a^ *p* value according to Linear Regression Model and Likelihood Ratio Test; significance level at *p* < 0.05.

^b^ *p* value is 0.413; the early stage (Tis‐ii) was used as the baseline characteristic for the analysis so it does not have an individual coefficient.

## 4 DISCUSSION

With improvements in survival, HNC survivors are living increasingly long posttreatment lives, often facing acute and chronic side effects from treatment. Understanding these outcomes in the overarching context of survivors’ lives is essential to understanding the impact of treatment and the significant impact on the day‐to‐day lives of survivors. Our study is the first to report flourishing in a clinical context, outside of mental health, examining the association of posttreatment symptoms on flourishing in cancer survivors to improve understanding of the relationship between symptoms and their effect on the lives of survivors. Our results show decreased flourishing is associated with dietary limitations, younger age, and lower income. Higher symptoms of depression, anxiety, swallowing dysfunction, neck disability, and insomnia along with reported financial difficulty was also associated with significant decreases in flourishing scores while controlling for age, sex, race, time since treatment, cancer stage and site, and education.

Our average SFI score is consistent with previously reported prepandemic population scores of 94.8 ± 29.8 reported by VanderWeele et al.^54^ Our analysis found a relationship between objective functional oral intake and flourishing. Those who had normal dietary intake reported higher flourishing scores than those who had limitations or tube‐dependent nutrition in overall flourishing scores and the subdomains of life satisfaction and mental and physical health. These results support prior research on the impact of swallowing dysfunction on relationships and quality‐of‐life measures but show that survivors perceive lower mental and physical health as well as overall life satisfaction and happiness when oral intake is restricted.^8^, ^55^, ^56^, ^57^ These results show that improving access to posttreatment swallowing evaluation may help improve not only weight and physical health but also their life satisfaction and happiness.^58^, ^59^

In addition to oral intake, socioeconomic variables lead to significant impacts on overall flourishing. Survivors who were employed showed higher overall flourishing scores than those who were on disability, retired, or unemployed. The impact of employment may be due to the social nature of work and the individual's involvement in their community. Income may also be a factor in how employment status affects flourishing, as those that reported lower incomes or difficulty paying for needs also reported lowers flourishing scores. The relationship between employment, income, and difficulty covering the costs of needs is complex and independent of the participant's education level. Notably, perceived difficulty paying for needs was significantly associated with all domains, while income and occupation were significantly associated across all domains except for domains for character and virtue and close social relationships. The stability of these domains in adversity supports the findings of the effects during the COVID19 pandemic, which show similar declines in character, virtue, and social relationships.^54^ Financial toxicity is well studied in cancer patients, with evidence that distress caused by high treatment costs impacts overall health.^30^, ^60^, ^61^ This study further supports reports on how economic barriers to health can affect outcomes and further shows how it may impact health‐related quality of life and flourishing. Our results suggest a need for financial reduction of financial toxicity to improve the lives of survivors.

Our study shows a correlation between patient reports of increasingly severe symptoms and lower flourishing scores (*p* < 0.001). Each PRO, including the PHQ8, GAD, EAT10, NDI, and ISI, showed an inverse relationship with flourishing. Prior research found an average 0.5 point decrease across all domains during the COVID‐19 pandemic compared with those before restrictions.^54^ Our results find that a one‐point increase was associated with at least a decrease of 0.76–2.64 points on the overall secure flourishing score for each outcome measured. When evaluating the scores considering MCID, all subdomains for depression, anxiety, and neck disability decreased by 0.5–2.9 points, suggesting that even mild increases in symptom severity are associated with significantly decreased flourishing. The impact of these outcomes on overall flourishing is likely multifactorial given the breadth of topics each questionnaire covers. It is known that disease and treatment contribute to anxiety and depression following diagnosis, affecting patients’ daily lives.^13^, ^14^, ^15^ The relationship between swallowing dysfunction and neck disability leads to limitations in social eating, driving, daily activities that may contribute to continued depression and anxiety, leading to the perception of lower quality of life and flourishing.^56^ We show that these physical and mental posttreatment outcomes have a significant effect on the relationships, meaning, and purpose of survivors. Survivors face numerous posttreatment outcomes, such as swallowing disorders, neck disability, depression, anxiety, and insomnia which require a team of experts familiar with head and neck carcinomas to help treat. Prior studies have highlighted the importance of establishing this care early.^62^, ^63^ Our results show treatment‐related toxicities are associated with a broad impact on the flourishing and well‐being of patients with additional research supporting that early intervention and management is not only to reduce symptom burden but also may be critical for the overall well‐being of patients.

### 4.1 Limitations

The survivors in this study are patients of a single institution's HNC survivorship clinic which focuses on the long‐term effects of treatment in posttreatment head and neck cancer patients and may limit generalizability. The cross‐sectional study design is limited by a single point in time rather than modeling changes in flourishing throughout treatment or with disease progression. Additionally, the limitation in sample size and diversity prevent us from examining the significant measures in more detail to understand further the relationships between race, staging, oral intake, and socioeconomic factors.

## 5 CONCLUSION

Understanding of what impacts overall life well‐being following treatment for head and neck cancer is becoming increasingly crucial for survivorship initiatives with the growing survivor population. Current research has focused on narrow variables, though this limits interpretation to narrow quality‐of‐life impacts. Our work shows that survivors who experience financial hardships or have limited oral intake are less likely to view themselves as living a good life. Additionally, common quality‐of‐life PROs with more negative responses are associated with lower flourishing scores, highlighting the importance of integration of care to reduce symptom burden and improve the overall well‐being of the survivors. Our results support the need for an integrated care model for posttreatment head and neck cancer survivors.

## ETHICS STATEMENT

University of Pittsburgh Institutional Review Board approved the study: STUDY20070027.

## CONFLICT OF INTEREST

The authors have no conflict of interest to declare.

## AUTHOR CONTRIBUTIONS

Alexandria Harris: Interviewed survivors, performed minor statistical analysis; interpreted results; prepared manuscript and submission. Jinhong Li: Designed the model, computational framework, and analyzed the data. Karley Atchison: Interviewed patients, prepared IRB submission, Supervised Data Collection. Christine Harrison: Interviewed patients, prepared IRB submission, Supervised Data Collection. Daniel Hall: Interpreted results, reviewed manuscript. Tyler VanderWeele: Interpreted results, reviewed manuscript, designed Flourishing Index. Jonas T. Johnson: Supervised Project. Marci L. Nilsen: Conceived of original idea, Interpreted results, Supervised Project.

## Data Availability

The data that support the findings of this study are available from the corresponding author upon request.
